# Sex-different interrelationships of rs945270, cerebral gray matter volumes, and attention deficit hyperactivity disorder: a region-wide study across brain

**DOI:** 10.1038/s41398-022-02007-8

**Published:** 2022-06-02

**Authors:** Xingguang Luo, Wenhua Fang, Xiandong Lin, Xiaoyun Guo, Yu Chen, Yunlong Tan, Leilei Wang, Xiaozhong Jing, Xiaoping Wang, Yong Zhang, Ting Yu, Jaime Ide, Yuping Cao, Lingli Yang, Chiang-Shan R. Li

**Affiliations:** 1 Beijing Huilongguan Hospital, Peking University Huilongguan Clinical School of Medicine, Beijing, 100096 China; 2 Department of Psychiatry, Yale University School of Medicine, New Haven, CT 06510 USA; 3 Department of Neurosurgery, The First Affiliated Hospital of Fujian Medical University, Fuzhou, Fujian 350001 China; 4 Laboratory of Radiation Oncology and Radiobiology, Fujian Medical University Cancer Hospital and Fujian Cancer Hospital, Fuzhou, 350014 China; 5 Shanghai Mental Health Center, Shanghai, 200030 China; 6 Department of Neurology, Shanghai Tongren Hospital, Shanghai Jiao Tong University, Shanghai, 200080 China; 7 Tianjin Mental Health Center, Tianjin, 300222 China; 8 Department of Psychiatry, the Second Xiangya Hospital, Central South University; The China National Clinical Research Center for Mental Health Disorders; National Technology Institute of Psychiatry; Key Laboratory of Psychiatry and Mental Health of Hunan Province, Changsha, 410017 China; 9 Department of Neuroscience, Yale University School of Medicine, New Haven, CT 06510 USA; 10 Wu Tsai Institute, Yale University, New Haven, CT 06510 USA

**Keywords:** Medical genetics, ADHD

## Abstract

Previous genome-wide association studies (GWAS) reported that the allele C of rs945270 of the kinectin 1 gene (*KTN1*) most significantly increased the gray matter volume (GMV) of the putamen and modestly regulated the risk for attention deficit hyperactivity disorder (ADHD). On the other hand, ADHD is known to be associated with a reduction in subcortical and cortical GMVs. Here, we examined the interrelationships of the GMVs, rs945270 alleles, and ADHD symptom scores in the same cohort of children. With data of rs945270 genotypes, GMVs of 118 brain regions, and ADHD symptom scores of 3372 boys and 3129 girls of the Adolescent Brain Cognition Development project, we employed linear regression analyses to examine the pairwise correlations adjusted for the third of the three traits and other relevant covariates, and examine their mediation effects. We found that the major allele C of rs945270 modestly increased risk for ADHD in males only when controlling for the confounding effects of the GMV of any one of the 118 cerebral regions (0.026 ≤ *p* ≤ 0.059: Top two: left and right putamen). This allele also significantly increased putamen GMV in males alone (left *p* = 2.8 × 10^−5^, and right *p* = 9.4 × 10^−5^; *α* = 2.1 × 10^−4^) and modestly increased other subcortical and cortical GMVs in both sexes (*α* < *p* < 0.05), whether or not adjusted for ADHD symptom scores. Both subcortical and cortical GMVs were significantly or suggestively reduced in ADHD when adjusted for rs945270 alleles, each more significantly in females (3.6 × 10^−7^ ≤ *p* < *α*; Top two: left pallidum and putamen) and males (3.5 × 10^−6^ ≤ *p* < *α*), respectively. Finally, the left and right putamen GMVs reduced 14.0% and 11.7% of the risk effects of allele C on ADHD, and allele C strengthened 4.5% (left) and 12.2% (right) of the protective effects of putamen GMVs on ADHD risk, respectively. We concluded that the rs945270-GMVs-ADHD relationships were sex-different. In males, the major allele C of rs945270 increased risk for ADHD, which was compromised by putamen GMVs; this allele also but only significantly increased putamen GMVs that then significantly protected against ADHD risk. In females, the top two GMVs significantly decreasing ADHD risk were left pallidum and putamen GMVs. Basal ganglia the left putamen in particular play the most critical role in the pathogenesis of ADHD.

## Introduction

The gray matter volumes (GMVs) of the basal ganglia (BG), including putamen [1, 2], pallidum [1–3] and caudate [4–6], have been consistently reported to be reduced in ADHD. Numerous studies have also demonstrated a reduction in cortical GMVs in ADHD, although the exact brain regions involved appeared to differ across studies, including the frontal [7–11], temporal [9], parietal [12], occipital [13], limbic [10], insular [11], and cerebellar [4, 14] cortices.

A previous genome-wide association study (GWAS) reported that the major allele C of rs945270 at 3’ flanking to the kinectin 1 gene (*KTN1*) showed the most significant positive effect on putamen GMV (*p* = 1.1 × 10^−33^) [15], a finding confirmed by follow-up meta GWAS (*p* = 1.0 × 10^−43^) [16] and candidate gene association studies (5.0 × 10^−51^ ≤ *p* ≤ 1.3 × 10^−5^) [17, 18]. This allele had also a significant positive effect on the pallium GMV (*p* = 3.0 × 10^−7^) [15]. That is, the major allele C of rs945270 was associated with higher GMVs of the BG. The *KTN1* encodes kinectin 1 receptor, which regulates neuronal cell shape and size [15, 19–21], and may thus play a critical role in determining regional brain volumes. This study focused on this most representative allele, although many genes might be involved in determining GMV, a multigenic complex trait.

The major allele C of rs945270 has also been reported to modestly reduce the symptom scores of ADHD (hyperactivity and inattention: 450 ≤ *n* ≤ 1834; 0.005 < *p* ≤ 0.057) [17, 22], serving as a possible protective factor for ADHD, consistent with the above positive “allele C–GMV” and negative “ADHD–GMV” associations. However, the alleles of nine other variants at the 5′- and 3′-UTRs of *KTN1* were reported to reliably increase both risks for ADHD and putamen GMV [23], which seemed to contradict the preceding negative “ADHD–GMV” associations. Further, it has been reported that the major allele C of rs945270 increased the risk for alcohol and nicotine co-dependence [24], a comorbid condition frequently observed in individuals with ADHD, and Parkinson’s disease [18, 25]. As the putamen GMV was significantly reduced in patients with alcoholism [26] and Parkinson’s disease [27–31], the latter seemed to contradict the afore-described finding of positive “allele C–GMV” associations, too. We posited that these contradictions probably resulted from mediation effects among these trait variables.

Notably, the earlier findings associating any pair of rs945270, GMVs, and ADHD were obtained in separate cohorts. For example, the rs945270-GMV association was examined only in the healthy populations [15]; rs945270-ADHD association was examined only in samples without GMV data [22]; and GMV–ADHD association was examined only in samples without rs945270 genotype data [32]. Thus, the potential mediation effects cannot be explored in these earlier studies. Here, aiming to disambiguate the interrelationships of the genetic and neural markers of ADHD, we examined how the *KTN1* alleles, cerebral GMVs, and ADHD symptom severity were related and potentially confounded in the same sample and whether these relationships differed between the sexes.

## Materials and methods

### Subjects

The Adolescent Brain Cognitive Development (ABCD) cohort comprises nearly 12,000 children (9–11 years old) enrolled from 21 sites across the country, including unrelated and related subjects (i.e., twins, triplets, and siblings) [33]. All participants were assessed with the Child Behavior Checklist for Ages 6–18 (CBCL/6–18) [34] and the T-scores of ADHD served as the phenotype. The ABCD project was approved by the Human Investigation Committee of all relevant institutions. All subjects’ parents or guardians signed written informed consents and children signed written assents prior to the study.

### Genetics

#### SNP genotyping, imputation, data cleaning, and power analysis

All ABCD subjects were genotyped using Affymetrix NIDA SmokeScreen Array, which, however, did not include the most representative rs945270. We imputed the untyped SNPs based on the 1000 Genome Project and HapMap3 Project data using the program IMPUTE2 [35], and filled in the genotype of rs945270 in ~70% of the unrelated subjects (3372 boys and 3129 girls). The imputed genotype data were stringently cleaned [36]. This sample had 80% of power to significantly (*α* = 0.05) differentiate a different rate of ADHD-T scores down to 9.7% (for boys) and 10.1% (for girls), respectively (analyzed by the R program “pwr.t.test”).

#### Zygosity inference

The ABCD subjects included unrelated subjects, siblings, and dizygotic and monozygotic twins or triplets. We inferred the genetic relationship (zygosity) between any pair of subjects using the whole genome data by the program PLINK [37]. In the present study, only unrelated subjects, whose relationships were confirmed both by self-report and genetic inference, were included. Related subjects and questionable subjects with unmatched genetic inference and self-report were excluded.

#### Estimation of admixture degree

To quantify the degree of admixture in these subjects, we estimated the ancestry proportions for each individual using the whole genome data by LD pruning [37] (see details in [36]) as implemented in the program STRUCTURE [38].

### Cortical and subcortical GMVs

Following published routines [39, 40], we implemented voxel-based morphometry (VBM) to quantify the GMVs of 52 cortical regions and seven subcortical structures as identified from high-resolution T1-weighted images with the CAT12 toolbox (http://dbm.neuro.uni-jena.de/vbm/). We evaluated the GMVs separately for the right and left hemispheric brain regions.

### Statistical analysis on the ADHD-rs945270-GMV relationship

ADHD-gene-GMV relationship is complicated because both ADHD traits and GMVs are multigenic and multifactorial, involving many genetic and environmental factors. To simplify the complexity of testing the interrelationships, we employ the symptom T-score to represent the severity of ADHD trait and the rs945270 marker to represent *KTN1*, but kept all 118 brain regions in the analysis. We evaluated the ADHD-rs945270-GMV relationship using the following two linear regression models as implemented in PLINK or SPSS 20.0. Males and females, and left and right hemispheric regions were analyzed separately.

#### ADHD ~ rs945270 + GMV + age + TIV + ancestry proportions (Model I)

In this polygenic model, the ADHD T-scores served as the dependent variable, the allele dosage of rs945270, GMV of one of the 118 brain regions, age, total intracranial volume (TIV), and the four dimensions of ancestry proportions served as independent variables. The four dimensions of ancestry proportions, corresponding to the four self-reported races of white, black, Hispanic and Asian, controlled for population stratification effects between different populations and admixture effects within each population.

The model allowed us to examine how rs945270 alleles predicted the risk for ADHD with the effects of GMVs and other covariates controlled for. Likewise, the model assessed how each of the 118 GMVs predicted the risk for ADHD with the potentially confounding effects of rs945270 and other covariates accounted for. As a contrast, we also conducted the analyses by removing the GMV (and TIV) and the rs945270 alleles from the models, respectively.

#### GMV ~ rs945270 + ADHD + age + TIV + ancestry proportions (Model II)

In this polygenic model, the GMV of one of the 118 brain regions served as the dependent variable, and the allele dosage of rs945270, ADHD T-scores, age, TIV, and the four dimensions of ancestry proportions served as independent variables. This model allowed us to examine how rs945270 alleles predicted the GMVs, with the potential confounding effects of ADHD symptom severity and other covariates controlled for, and how ADHD T-scores predicted the GMVs, with the effects of rs945270 and other covariates controlled for. As a contrast, we also conducted the analyses by removing the ADHD T-scores and rs945270 from the models, respectively.

The significance level (α) for the correlation was corrected by the numbers of brain regions (*n* = 118) and analytic models (*n* = 2), and thus set at 2.1 × 10^−4^.

#### Mediation analysis

To examine the mediation effects of GMVs on the associations between ADHD-T scores and rs945270, we followed three steps as integrated in Model I and II. Step 1 was a linear regression analysis on the model “ADHD ~ α1*rs945270 + age + ancestry proportions”, in which ADHD-T scores served as the dependent variable, the allele dosage of rs945270, age, and the four dimensions of ancestry proportions served as independent variables, and α1 was the regression coefficient for rs945270. If the effect of rs945270 was significant, we went to the next step. Step 2 was a linear regression on the model “GMV ~ β1*rs945270 + age + TIV + ancestry proportions”, in which GMV was the putative mediator and β1 was the regression coefficient for rs945270. When the effect of rs945270 was significant, we went to next step. Step 3 was a linear regression on the model “ADHD ~ α2*rs945270 + β2*GMV + age + TIV + ancestry proportions”, in which α2 and β2 were the regression coefficients for rs945270 and GMV, respectively. If the effect of GMV (β2) was significant, we claimed that the GMV significantly mediated the association between ADHD and rs945270.

Similarly, to examine the potential mediation effects of rs945270 on the associations between ADHD-T scores and GMVs, we followed these three steps: Step 1 (linear regression): “ADHD ~ α1*GMV + TIV + age + ancestry proportions”; Step 2 (logistic regression): “rs945270 ~ β1*GMV + age + TIV + ancestry proportions”; and Step 3 (linear regression): “ADHD ~ α2*GMV + β2*rs945270 + age + TIV + ancestry proportions”. Only when *p* values for α1, β1 and β2 were all significant, we claimed a significant mediation effect.

## Results

### The major allele C of rs945270 significantly increased risk for ADHD in males, particularly when adjusted for BG GMVs

**Fig. 1:**
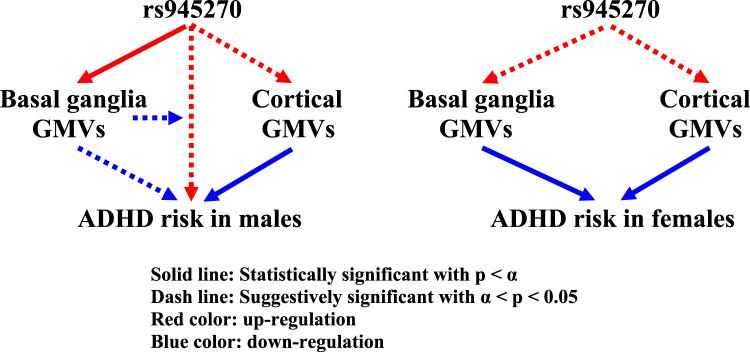
Regulation pathways for rs945270, GMVs, and ADHD risk. [In males, the major allele C of rs945270 significantly (*p* < *α*) and suggestively (*α* < *p* < 0.05) upregulated the basal ganglia and cortical GMVs, respectively (red); the basal ganglia and cortical GMVs suggestively and significantly downregulated ADHD risk, respectively (blue); and the allele C suggestively upregulated ADHD risk, which was repressed by basal ganglia GMVs. In females, the allele C suggestively upregulated the basal ganglia and cortical GMVs (red); and the basal ganglia and cortical GMVs significantly downregulated ADHD risk (blue).].

Before adjustment for TIV and GMVs, the results of Model I showed that the association between ADHD T-scores and the major allele C of rs945270 was suggestively significant in males (*β* = 0.291; *p* = 0.056) but not in females (*β* = 0.039; *p* = 0.763). After adjustment for TIV and GMVs, these associations became significant or suggestively significant in males (all *β* > 0; 0.026 ≤ *p* ≤ 0.059 for all 118 brain regions; data not shown) but remained insignificant in females (*p* ≥ 0.478). The major allele C trended with higher ADHD T-scores (all *β* > 0) in males, and the top significant ADHD-rs945270 associations were observed when BG GMVs served as covariates [putamen (left: *p* = 0.026, and right: *p* = 0.030), pallidum (left: *p* = 0.031, and right: *p* = 0.034), and caudate (left: *p* = 0.040, and right: *p* = 0.040)] (Fig. 1).

### The major allele C of rs945270 significantly increased the GMVs, particularly putamen GMV in males (Fig. 1)

The results of Model II showed that the only significant (*α* = 2.1 × 10^−4^) associations between rs945270 allele C and GMV were observed for the putamen (left: *β* = 0.005, *p* = 2.8 × 10^−5^; and right: *β* = 0.005, *p* = 9.4 × 10^−5^) in males. Pallidum GMV and the major allele C were also correlated but slightly short of significance (left: *β* = 0.004, *p* = 1.9 × 10^−3^; and right: *β* = 0.003, *p* = 4.1 × 10^−3^) in males. These four associations were only nominally significant (*α* < *p* < 0.05) but also ranked at the top four in females (Table 1). The major allele C was also nominally (*α* < *p* < 0.05) associated with the GMVs of 22 other regions (Supplementary Table S1). None of the nominal associations survived the Bonferroni correction for multiple testing (*α* = 2.1 × 10^−4^), though all trending in the same direction (*β* > 0). Finally, the significance levels and directions of association did not change significantly if the ADHD T-scores were not adjusted for (Table 1).

**Table 1:** Associations between GMVs and major allele C of rs945270 in males and females.

		Left putamen		Right putamen		Left pallidum		Right pallidum	
	Covariate	*β*	*p*	*β*	*p*	*β*	*p*	*β*	*p*
Male	With ADHD-T	5.1 × 10^−3^	**2.8** × **10**^**−5**^	4.6 × 10^−3^	**9.4** × **10**^**−5**^	4.3 × 10^−3^	1.9 × 10^−3^	3.1 × 10^−3^	4.1 × 10^−3^
	Without ADHD-T	5.2 × 10^−3^	**2.6** × **10**^**−5**^	4.5 × 10^−3^	**1.7** × **10**^**−4**^	4.1 × 10^−3^	3.2 × 10^−3^	3.0 × 10^−3^	6.0 × 10^−3^
Female	With ADHD-T	4.0 × 10^−3^	8.0 × 10^−4^	3.5 × 10^−3^	3.1 × 10^−3^	4.4 × 10^−3^	1.0 × 10^−3^	3.0 × 10^−3^	5.2 × 10^−3^
	Without ADHD-T	4.2 × 10^−3^	6.5 × 10^−4^	3.4 × 10^−3^	3.9 × 10^−3^	4.3 × 10^−3^	1.5 × 10^−3^	2.9 × 10^−3^	6.8 × 10^−3^

Bold: *p* < α = 2.1 × 10^−4^. Other nominal associations with *p* < 0.05 are listed in Supplementary Table S1.

### ADHD was significantly or suggestively associated with reduction of cortical and subcortical GMVs in males and females (Fig. 1)

Both Models I and II showed that ADHD T-scores were significantly associated with reduction (*β* < 0) of cortical and subcortical GMVs in males and females.

In males, both Models I and II showed that ADHD T-scores were significantly (*α* = 2.1 × 10^−4^) associated with reduction of GMVs of frontal (left and right inferior orbital, right middle, left and right middle orbital, left superior, left and right superior orbital, and left rectus), parietal (left supramarginal), and left insular cortices (3.5 × 10^−6^ ≤ *p* ≤ 2.1 × 10^−4^, Model I), when adjusting for rs945270 (Table 2). Furthermore, if not adjusting for rs945270, Models I and II derived the same *p* values (4.0 × 10^−10^ ≤ *p* ≤ 3.0 × 10^−5^; Table 2); however, these *p* values were much smaller than those derived from the analyses with adjustment for rs945270 (Table 2), but all association directions remained the same. Model II also showed that the ADHD T-score was significantly associated with reduction of GMV of left olfactory (*p* = 1.5 × 10^−4^) and left rectus (*p* = 1.5 × 10^−4^) gyrus (Supplementary Table S2). In addition, ADHD T-scores were also nominally (*α* ≤ *p* < 0.05) associated with reduction of GMVs of 80 other cortical and subcortical regions, including the BG [putamen (left: 3.1 × 10^−4^ ≤ *p* ≤ 0.001, and right: 0.003 ≤ *p* ≤ 0.009), pallidum (left: 7.4 × 10^−4^ ≤ *p* ≤ 0.003, and right: 0.006 ≤ *p* ≤ 0.015), caudate head (right: *p* = 0.001, and left: *p* = 0.009), and entire caudate (right: *p* = 0.004, and left: 0.016 ≤ *p* ≤ 0.019)] (Table 2; Supplementary Table S2).

**Table 2:** Significant associations between GMVs and ADHD in males.

	Adjusted for rs945270		Not adjusted for rs945270	
	Model I	Model II	Model I	Model II
Region	*p*	*p*	*p*	*p*
Frontal (right inferior orbital) cortex	3.5 × 10^−6^	2.7 × 10^−5^	4.0 × 10^−10^	4.0 × 10^−10^
Frontal (left inferior orbital) cortex	1.1 × 10^−5^	7.1 × 10^−5^	7.9 × 10^−9^	7.9 × 10^−9^
Frontal (left superior) cortex	1.3 × 10^−5^	3.8 × 10^−5^	1.0 × 10^−6^	1.0 × 10^−6^
Frontal (right superior orbital) cortex	5.9 × 10^−6^	9.3 × 10^−6^	5.6 × 10^−9^	5.6 × 10^−9^
Frontal (left superior orbital) cortex	2.3 × 10^−5^	4.3 × 10^−5^	2.1 × 10^−7^	2.1 × 10^−7^
Frontal (right middle orbital) cortex	6.3 × 10^−5^	2.0 × 10^−4^	2.3 × 10^−8^	2.3 × 10^−8^
Frontal (right middle) cortex	1.1 × 10^−4^	1.6 × 10^−4^	2.0 × 10^−6^	2.0 × 10^−6^
Frontal (left middle orbital) cortex	2.1 × 10^−4^	*4.1* × *10*^*−4*^	1.6 × 10^−7^	1.6 × 10^−7^
Left supramarginal cortex	4.5 × 10^−5^	3.1 × 10^−5^	4.0 × 10^−6^	4.0 × 10^−6^
Left insula	2.5 × 10^−5^	3.4 × 10^−5^	6.0 × 10^−6^	6.0 × 10^−6^
Left rectus	4.9 × 10^−5^	3.1 × 10^−5^	3.0 × 10^−5^	3.0 × 10^−5^
Left putamen	*0.001*	*3.1* × *10*^*−4*^	4.6 × 10^−5^	4.6 × 10^−5^
Right putamen	*0.009*	*0.003*	*2.7* × *10*^*−4*^	*2.7* × *10*^*−4*^

All *β* < 0. Italic: *p* > *α* = 2.1 × 10^−4^. Model I: “ADHD~GMV + covariates”; Model II: “GMV~ADHD + covariates”. Other nominal associations in males are listed in Supplementary Table S2.

In females, both Models I and II showed that ADHD T-scores were significantly (*α* = 2.1 × 10^−4^) associated with reduction of BG GMVs [putamen (left: *p* = 1.8 × 10^−6^), pallidum (left: *p* = 3.6 × 10^−7^, and right: *p* = 1.5 × 10^−5^), caudate head (left: *p* = 2.4 × 10^−5^, and right: *p* = 4.3 × 10^−5^), and entire caudate (left: *p* = 4.7 × 10^−5^, and right: 6.9 × 10^−5^); Model I], and eight cortical regions [right inferior parietal, left middle occipital, right inferior temporal, left and right middle temporal, left fusiform, right angular and left cerebelum_6 (1.4 × 10^−5^ ≤ *p* ≤ 1.6 × 10^−4^; Model I)], when adjusting for rs945270 (Table 3). Furthermore, if not adjusting for rs945270, Models I and II derived the same *p* values and kept the association directions; however, these *p* values were a little bit altered for cortical regions (4.1 × 10^−6^ ≤ *p* ≤ 2.8 × 10^−4^), but became much larger for BG (3.5 × 10^−5^ ≤ *p* ≤ 4.7 × 10^−4^), as compared to models with adjustment for rs945270 (Table 3). In addition, the ADHD T-scores were also nominally (*α* ≤ *p* < 0.05) associated with the reduction of GMVs of 85 other cortical and subcortical regions (Supplementary Table S3). As the only exception, both models showed that the ADHD T-scores were nominally associated with an enlargement (*β* > 0) of GMVs of the cerebellar vermis, section 3 (0.016 ≤ *p* ≤ 0.021) (Supplementary Table S3).

**Table 3:** Significant associations between GMVs and ADHD in females.

	Adjusted for rs945270		Not adjusted for rs945270	
	Model I	Model II	Model I	Model II
Region	*p*	*p*	*p*	*p*
Left putamen	1.8 × 10^−6^	1.7 × 10^−6^	*2.4* × *10*^*−4*^	*2.4* × *10*^*−4*^
Right putamen	*4.0* × *10*^*−4*^	*8.8* × *10*^*−4*^	*7.2* × *10*^*−3*^	*7.2* × *10*^*−3*^
Left pallidum	3.6 × 10^−7^	3.1 × 10^−7^	3.5 × 10^−5^	3.5 × 10^−5^
Right Pallidum	1.5 × 10^−5^	4.5 × 10^−5^	*4.7* × *10*^*−4*^	*4.7* × *10*^*−4*^
Left caudate head	2.4 × 10^−5^	3.0 × 10^−5^	8.9 × 10^−5^	8.9 × 10^−5^
Right caudate head	4.3 × 10^−5^	1.2 × 10^−4^	4.5 × 10^−5^	4.5 × 10^−5^
Left caudate	4.7 × 10^−5^	5.0 × 10^−5^	1.8 × 10^−4^	1.8 × 10^−4^
Right caudate	6.9 × 10^−5^	1.7 × 10^−4^	9.4 × 10^−5^	9.4 × 10^−5^
Right inferior parietal	5.0 × 10^−5^	*3.0* × *10*^*−4*^	1.4 × 10^−5^	1.4 × 10^−5^
Left middle occipital	1.1 × 10^−4^	*3.9* × *10*^*−4*^	4.0 × 10^−6^	4.0 × 10^−6^
Right inferior temporal	2.4 × 10^−5^	3.8 × 10^−5^	9.6 × 10^−5^	9.6 × 10^−5^
Left middle temporal	3.6 × 10^−5^	9.7 × 10^−5^	1.7 × 10^−5^	1.7 × 10^−5^
Right middle temporal	6.3 × 10^−5^	1.8 × 10^−4^	2.3 × 10^−5^	2.3 × 10^−5^
Left fusiform	1.1 × 10^−4^	*2.3* × *10*^*−4*^	7.3 × 10^−5^	7.3 × 10^−5^
Right angular	1.6 × 10^−4^	*6.1* × *10*^*−4*^	*2.8* × *10*^*−4*^	*2.8* × *10*^*−4*^
Left cerebelum_6	1.4 × 10^−5^	2.0 × 10^−5^	1.9 × 10^−5^	1.9 × 10^−5^

All *β* < 0 and all *p* < *α* = 2.1 × 10^−4^ except for the italic. Models I and II: same as Table 2. Other nominal associations in females are listed in Supplementary Table S3.

### Allele C of rs945270 strengthened the protective effects of putamen GMVs on ADHD and putamen GMVs weakened the risk effects of allele C on ADHD in males

In the analysis on the mediation effects of GMVs on the ADHD-rs945270 associations, Step 1 showed that the association between ADHD T-scores and the major allele C of rs945270 was suggestively significant in males (*α*1 = 0.291; *p* = 0.056) (see also Section 3.1). Step 2 showed that the only significant associations were between rs945270 allele C and putamen GMVs (left: *β*1 = 0.005, *p* = 2.6 × 10^−5^; and right: *β*1 = 0.005, *p* = 1.7 × 10^−4^) in males (see also Section 3.2; Table 1). Step 3 showed significant effects of rs945270 (left: *α*2 = 0.338, *p* = 0.026; and right: *α*2 = 0.330, *p* = 0.030; see also Section 3.1) and putamen GMVs (left: *β*2 = −6.845, *p* = 0.001; and right: *β*2 = −5.758, *p* = 0.009; Table 2) on ADHD in males. No other significant mediation effects of GMVs can be claimed either in males or females.

In the analysis on the mediation effects of rs945270 on the ADHD-GMV associations, Step 1 showed that the association between ADHD T-scores and putamen GMVs was significant in males (left: *α*1 = −7.166, *p* = 4.6 × 10^−5^; and right: *α*1 = −6.558, *p* = 2.7 × 10^−4^) (Table 2). Step 2 showed the only significant associations were between allele C of rs945270 and putamen GMVs (left: *β*1 = 1.035, *p* = 2.7 × 10^−5^; and right: *β*1 = 0.970, *p* = 1.3 × 10^−4^) in males (data not shown). Step 3 showed significant effects of putamen GMVs (left: *α*2 = −6.845, *p* = 0.001; and right: *α*2 = −5.758, *p* = 0.009; see also Table 2) and allele C (left: *β*2 = 0.338, *p* = 0.026; and right: *β*2 = 0.330, *p* = 0.030; see also Section 3.1) on ADHD in males. No other significant mediation effects of rs945270 can be claimed either in males or females.

## Discussion

We demonstrated that the rs945270-GMVs-ADHD relationships were sex-different. In males, the major allele C of rs945270 increased the risk for ADHD, and this risk effect was mitigated by putamen GMVs; this allele also but only significantly increased putamen GMVs that then significantly protected against ADHD risk. In females, the top two GMVs significantly decreasing ADHD risk were left pallidum and putamen GMVs. These findings support that GMVs of the putamen, particularly the left putamen, played the most critical role in the pathogenesis of ADHD.

Specifically, the major allele C of rs945270 modestly increased the risk for ADHD in males only when controlling for the confounding effects of any one of 118 cerebral regional GMVs, most significantly among which were the left and right putamen GMVs. If the confounding effect of any GMV was not controlled for, the rs945270-ADHD association turned to be non-significant, as also demonstrated in Psychiatric Genomics Consortium (PGC) data [41] and previous GWAS [42]. However, in females, the rs945270-ADHD association was not significant, even after adjusting for GMVs. Furthermore, this allele also significantly increased putamen GMV only in males, confirming previous findings [15, 17, 18]. The regulatory effect of the major allele C of rs945270 on GMVs was not influenced by ADHD symptom scores. In females, of all 118 regions the putamen GMV also ranked Top 1 that were increased by allele C, though not statistically significantly whether adjusted for ADHD symptom scores or not.

The GMVs of many cortical and subcortical regions were significantly reduced in ADHD in both sexes. In males, the most significant ADHD-related GMV reduction was observed for the frontal (eight regions), left supramarginal, insular cortices, and left gyrus rectus, which were reduced only at suggestive significance in females. In females, the most significant ADHD-related GMV reduction was observed for the BG (putamen, pallidum and caudate), and two parietal, one occipital, four temporal cortical, and one cerebellar, regions, which were only suggestively significant in males. Higher cortical GMVs significantly protected against ADHD risk in both sexes, in accord with previous findings [4, 7–14]. The finding that BG GMVs protected against ADHD risk was also consistent with numerous previous reports [1–6]. If the confounding effects of rs945270 were not controlled for, both cortical and BG GMV–ADHD associations became much more significant in males; however, cortical GMV–ADHD associations were slightly altered and BG GMV–ADHD associations, especially for left putamen and pallidum, became much less significant in females. These findings together suggest that, in male allele C carriers, BG GMV enlarged significantly and exerted a significant protective effect on ADHD risk, which explained why the allele C strengthened BG GMV–ADHD associations significantly in males. In contrast, in female allele C carriers, BG GM enlarged modestly and exerted only a limited protective effect on ADHD risk, which explained why the allele C weakened BG GMV–ADHD associations significantly and why the change of the overall ADHD risk in allele C carriers did not reach statistical significance in females.

To explore the potential mediation effects of putamen GMVs underlying the risk effects of rs945270 on ADHD, we performed a mediation analysis that showed a larger direct effect (*α*2) than total effect (*α*1) of allele C of rs945270 on ADHD risk (i.e., *α*2 > *α*1), indicating an indirect, protective effect (*α*1−*α*2 < 0) of putamen GMVs underlying the association between ADHD and rs945270 in males. In support, the left and right putamen GMVs reduced 14.0% and 11.7% [=|*α*2−*α*1|/*α*2] of the risk effects of allele C on ADHD, respectively.

Regarding the mediation of allele C of rs945270 of the protective effect of putamen GMVs on ADHD, the findings showed a larger and much more significant total protective effect (*α*1) than the direct protective effect (*α*2) of putamen GMVs on ADHD (i.e., *α*1 < 0, *α*2 < 0, and |*α*1| > |*α*2|), indicating an indirect protective effect (*α*1−*α*2 < 0) between putamen GMVs and ADHD as mediated through allele C of rs945270, in males. This indirect effect measured the amount of mediation, reflecting the protective effect of putamen GMVs on ADHD due to allele C. In support, allele C of rs945270 strengthened 4.5% (left) and 12.2% (right) [=(|*α*1 − *α*2|)/*α*1] of the protective effects of putamen GMVs on ADHD risk, respectively. This also suggests that a larger regulatory effect of allele C on putamen GMVs that protects against ADHD risk than its regulatory effect on ADHD that increases ADHD risk, resulting in an overall diminished risk, as observed previously [17, 22]. In contrast, if an allele has a smaller regulatory effect on putamen GMVs than its regulatory effect on ADHD, the overall risk effect would be positive, as in previous reports that nine other *KTN1* alleles reliably increased risk for ADHD [23] and allele C of rs945270 increased risk for alcohol and nicotine co-dependence [24], comorbidity of ADHD.

In summary, the primary regulation target of rs945270 was putamen GMV, which served as the main depressor of the risk effect of allele C of rs945270 on ADHD in males; and allele C was the primary enhancer for the protective effect of putamen GMVs on ADHD in males; putamen GMVs may play the most critical role in the pathogenesis of ADHD in both sexes.

## Supplementary information

Supplementary Table S1

Supplementary Table S2

Supplementary Table S3
