# Supplementary material for: Sex-different interrelationships of rs945270, cerebral gray matter volumes, and attention deficit hyperactivity disorder: a region-wide study across brain
Source: Transl Psychiatry. 2022 Jun 2;12:225. doi: 10.1038/s41398-022-02007-8 (PMC9163172; doi:10.1038/s41398-022-02007-8)
Supplement: Supplementary file 1 — Supplementary Table S1 [file 41398_2022_2007_MOESM1_ESM.doc]

**Table S1. Nominal associations between GMVs and major allele C of rs945270 in females**

|  | Male | | Female | |
| --- | --- | --- | --- | --- |
| Region | β | p | β | p |
| Right temporal pole | 0.003 | 0.026 |  |  |
| Right middle temporal cortex | 0.002 | 0.030 |  |  |
| Left inferior temporal cortex | 0.002 | 0.037 |  |  |
| Right precentral cortex | 0.002 | 0.044 | 0.002 | 0.026 |
| Left insula | 0.002 | 0.050 |  |  |
| Left angular cortex |  |  | 0.003 | 0.014 |
| Right caudate |  |  | 0.003 | 0.015 |
| Right cerebelum_10 |  |  | 0.001 | 0.032 |
| Left cerebellum crus 1 cortex |  |  | 0.002 | 0.044 |
| Left cuneus |  |  | 0.004 | 0.002 |
| Right cuneus |  |  | 0.003 | 0.007 |
| Right superior frontal cortex |  |  | 0.002 | 0.026 |
| Right fusiform |  |  | 0.003 | 0.018 |
| Left lingual cortex |  |  | 0.002 | 0.041 |
| Left inferior occipital cortex |  |  | 0.003 | 0.010 |
| Right middle occipital cortex |  |  | 0.002 | 0.030 |
| Left paracentral lobule |  |  | 0.002 | 0.044 |
| Right paracentral lobule |  |  | 0.002 | 0.044 |
| Left superior parietal cortex |  |  | 0.002 | 0.010 |
| Left precuneus |  |  | 0.003 | 0.016 |
| Right precuneus |  |  | 0.003 | 0.013 |
| Right superior motor area |  |  | 0.003 | 0.013 |

All p>α=2.1×10^-4^ and all β>0. The blank cells correspond to p>0.05.
