# Supplementary material for: Sex-different interrelationships of rs945270, cerebral gray matter volumes, and attention deficit hyperactivity disorder: a region-wide study across brain
Source: Transl Psychiatry. 2022 Jun 2;12:225. doi: 10.1038/s41398-022-02007-8 (PMC9163172; doi:10.1038/s41398-022-02007-8)
Supplement: Supplementary file 2 — Supplementary Table S2 [file 41398_2022_2007_MOESM2_ESM.doc]

**Table S2. P values for nominal associations between GMVs and ADHD in males**

|  | Model I | Model II |  | Model I | Model II |
| --- | --- | --- | --- | --- | --- |
| Region | p | p | Region | p | p |
| Pallidum_L | 0.003 | 7.4×10^-4^ | Precuneus_R | 0.038 | 0.054 |
| Pallidum_R | 0.015 | 0.006 | Calcarine_L | 0.018 | 0.027 |
| Caudate_Head_L | 0.009 | 0.009 | Cuneus_L | 0.022 | 0.034 |
| Caudate_Head_R | 0.001 | 0.001 | Cuneus_R | 0.019 | 0.031 |
| Caudate_L | 0.019 | 0.016 | Lingual_L | 0.004 | 0.009 |
| Caudate_R | 0.004 | 0.004 | Lingual_R | 0.043 | 0.070 |
| Thalamus_L | 0.004 | 0.005 | Occipital_Inf_L | 0.029 | 0.041 |
| Thalamus_R | 0.014 | 0.016 | Occipital_Inf_R | 0.004 | 0.005 |
| Amygdala_L | 5.8×10^-4^ | 4.8×10^-4^ | Occipital_Mid_L | 5.7×10^-4^ | 8.2×10^-4^ |
| Amygdala_R | 0.016 | 0.015 | Occipital_Mid_R | 2.3×10^-4^ | 4.4×10^-4^ |
| Cingulum_Ant_L | 0.033 | 0.035 | Occipital_Sup_L | 8.8×10^-4^ | 1.4×10^-3^ |
| Cingulum_Ant_R | 0.033 | 0.028 | Occipital_Sup_R | 0.006 | 0.013 |
| Cingulum_Mid_R | 0.023 | 0.025 | Fusiform_L | 0.013 | 0.016 |
| Cingulum_Post_L | 0.003 | 0.007 | Fusiform_R | 0.011 | 0.022 |
| Cingulum_Post_R | 0.014 | 0.019 | Heschl_L | 0.037 | 0.023 |
| Hippocampus_L | 7.7×10^-4^ | 4.2×10^-4^ | Heschl_R | 0.013 | 0.008 |
| Hippocampus_R | 0.009 | 0.007 | Temporal_Inf_L | 0.001 | 0.002 |
| ParaHippocampal_L | 0.015 | 0.016 | Temporal_Inf_R | 0.003 | 0.005 |
| ParaHippocampal_R | 0.016 | 0.017 | Temporal_Mid_L | 0.016 | 0.021 |
| Frontal_Inf_Oper_R | 0.049 | 0.091 | Temporal_Mid_R | 0.001 | 0.003 |
| Frontal_Inf_Tri_L | 5.7×10^-4^ | 9.3×10^-4^ | Temporal_Pole_Sup_L | 0.009 | 0.019 |
| Frontal_Inf_Tri_R | 0.002 | 0.004 | Temporal_Pole_Sup_R | 0.036 | 0.050 |
| Frontal_Med_Orb_L | 7.0×10^-4^ | 1.3×10^-3^ | Temporal_Sup_L | 4.0×10^-4^ | 4.1×10^-4^ |
| Frontal_Med_Orb_R | 5.5×10^-4^ | 1.0×10^-3^ | Insula_R | 0.003 | 0.005 |
| Frontal_Mid_L | 0.002 | 0.002 | Olfactory_L | 5.8×10^-4^ | **1.5×10^-4^** |
| Frontal_Sup_Medial_L | 4.9×10^-4^ | 8.0×10^-4^ | Olfactory_R | 0.002 | 4.5×10^-4^ |
| Frontal_Sup_Medial_R | 0.004 | 0.007 | Cerebelum_10_L | 0.006 | 0.006 |
| Frontal_Sup_R | 0.002 | 0.004 | Cerebelum_7b_L | 0.037 | 0.085 |
| Precentral_R | 0.031 | 0.030 | Cerebelum_7b_R | 0.006 | 0.019 |
| Rolandic_Oper_L | 0.014 | 0.008 | Cerebelum_8_L | 0.002 | 0.007 |
| Rolandic_Oper_R | 0.001 | 9.1×10^-4^ | Cerebelum_8_R | 0.017 | 0.038 |
| Supp_Motor_Area_L | 0.023 | 0.027 | Cerebelum_9_L | 0.018 | 0.029 |
| Supp_Motor_Area_R | 0.025 | 0.039 | Cerebelum_Crus1_L | 0.004 | 0.010 |
| Angular_R | 8.9×10^-4^ | 1.6×10^-3^ | Cerebelum_Crus1_R | 0.015 | 0.038 |
| Parietal_Inf_L | 0.002 | 0.003 | Cerebelum_Crus2_L | 0.046 | 0.103 |
| Parietal_Inf_R | 0.002 | 0.003 | Cerebelum_Crus2_R | 0.041 | 0.087 |
| Parietal_Sup_L | 0.010 | 0.012 | Rectus_R | 2.3×10^-4^ | **1.5×10^-4^** |
| Parietal_Sup_R | 6.1×10^-4^ | 8.9×10^-4^ | Vermis_4_5 | 0.052 | 0.041 |
| Precuneus_L | 0.005 | 0.008 | Vermis_8 | 0.031 | 0.059 |

All p>α=2.1×10^-4^ but left olfactory and right rectus (**Bold**), and all β<0; with adjustment for rs945270. Models I and II: same as Table 2. L, left; R, right; Ant, anterior; Post, posterior; Inf, inferior; Mid, middle; Sup, superior; Oper, pars opercularis; Tri, pars triangularis; Orb, orbital; Supp, supplementary.
