# Supplementary material for: Sex-different interrelationships of rs945270, cerebral gray matter volumes, and attention deficit hyperactivity disorder: a region-wide study across brain
Source: Transl Psychiatry. 2022 Jun 2;12:225. doi: 10.1038/s41398-022-02007-8 (PMC9163172; doi:10.1038/s41398-022-02007-8)
Supplement: Supplementary file 3 — Supplementary Table S3 [file 41398_2022_2007_MOESM3_ESM.doc]

**Table S3. P values for nominal associations between GMVs and ADHD in females**

|  | Model I | Model II |  | Model I | Model II |
| --- | --- | --- | --- | --- | --- |
| Region | p | p | Region | p | p |
| Caudate_R | 2.2×10^-4^ | 4.6×10^-4^ | Precuneus_R | 0.009 | 0.009 |
| Amygdala_L | 0.004 | 0.002 | SupraMarginal_L | 0.022 | 0.050 |
| Amygdala_R | 0.010 | 0.009 | SupraMarginal_R | 2.4×10^-4^ | 8.0×10^-4^ |
| Cingulum_Ant_L | 0.008 | 0.017 | Calcarine_L | 0.002 | 0.003 |
| Cingulum_Ant_R | 0.007 | 0.020 | Calcarine_R | 0.015 | 0.011 |
| Cingulum_Mid_L | 0.017 | 0.018 | Cuneus_L | 0.008 | 0.010 |
| Cingulum_Mid_R | 0.004 | 0.006 | Cuneus_R | 0.016 | 0.020 |
| Hippocampus_L | 6.1×10^-4^ | 7.8×10^-4^ | Lingual_L | 0.002 | 0.002 |
| Hippocampus_R | 0.014 | 0.021 | Lingual_R | 0.017 | 0.021 |
| ParaHippocampal_L | 0.001 | 0.003 | Occipital_Inf_L | 0.005 | 0.011 |
| ParaHippocampal_R | 0.016 | 0.027 | Occipital_Inf_R | 0.012 | 0.028 |
| Frontal_Inf_Oper_L | 0.002 | 0.010 | Occipital_Mid_R | 0.017 | 0.030 |
| Frontal_Inf_Oper_R | 4.5×10^-4^ | 0.003 | Occipital_Sup_L | 0.002 | 0.007 |
| Frontal_Inf_Orb_L | 0.011 | 0.094 | Occipital_Sup_R | 0.011 | 0.014 |
| Frontal_Inf_Orb_R | 0.039 | 0.167 | Fusiform_R | 4.2×10^-4^ | 0.001 |
| Frontal_Inf_Tri_L | 0.002 | 0.010 | Heschl_L | 0.025 | 0.041 |
| Frontal_Inf_Tri_R | 0.008 | 0.023 | Heschl_R | 0.013 | 0.016 |
| Frontal_Med_Orb_L | 0.037 | 0.121 | Temporal_Inf_L | 0.003 | 0.004 |
| Frontal_Med_Orb_R | 0.003 | 0.012 | Temporal_Pole_Mid_L | 0.001 | 0.003 |
| Frontal_Mid_L | 0.002 | 0.006 | Temporal_Pole_Mid_R | 0.001 | 0.001 |
| Frontal_Mid_Orb_L | 0.010 | 0.029 | Temporal_Pole_Sup_L | 0.001 | 0.005 |
| Frontal_Mid_Orb_R | 0.014 | 0.057 | Temporal_Pole_Sup_R | 0.016 | 0.038 |
| Frontal_Mid_R | 0.004 | 0.017 | Temporal_Sup_L | 0.003 | 0.011 |
| Frontal_Sup_L | 0.035 | 0.140 | Temporal_Sup_R | 3.7×10^-4^ | 7.9×10^-4^ |
| Frontal_Sup_Medial_L | 0.012 | 0.029 | Insula_L | 0.002 | 0.006 |
| Frontal_Sup_Medial_R | 0.019 | 0.035 | Insula_R | 0.002 | 0.004 |
| Frontal_Sup_Orb_L | 0.028 | 0.074 | Cerebelum_4_5_L | 4.4×10^-4^ | 3.0×10^-4^ |
| Frontal_Sup_Orb_R | 0.009 | 0.031 | Cerebelum_4_5_R | 0.025 | 0.024 |
| Frontal_Sup_R | 0.016 | 0.060 | Cerebelum_6_R | 0.003 | 0.003 |
| Precentral_L | 9.1×10^-4^ | 0.005 | Cerebelum_7b_L | 6.1×10^-4^ | 0.002 |
| Precentral_R | 0.004 | 0.013 | Cerebelum_7b_R | 0.003 | 0.013 |
| Rolandic_Oper_L | 0.006 | 0.017 | Cerebelum_8_L | 0.003 | 0.007 |
| Rolandic_Oper_R | 7.4×10^-4^ | 0.002 | Cerebelum_8_R | 0.047 | 0.088 |
| Supp_Motor_Area_L | 0.005 | 0.006 | Cerebelum_9_L | 0.030 | 0.029 |
| Supp_Motor_Area_R | 0.008 | 0.012 | Cerebelum_Crus1_L | 3.2×10^-4^ | 8.2×10^-4^ |
| Angular_L | 0.005 | 0.011 | Cerebelum_Crus1_R | 0.001 | 0.003 |
| Parietal_Inf_L | 0.002 | 0.005 | Cerebelum_Crus2_L | 0.012 | 0.040 |
| Parietal_Sup_L | 0.037 | 0.053 | Cerebelum_Crus2_R | 0.004 | 0.015 |
| Parietal_Sup_R | 0.007 | 0.008 | Rectus_L | 0.040 | 0.093 |
| Postcentral_L | 0.001 | 0.001 | Rectus_R | 0.025 | 0.064 |
| Postcentral_R | 0.009 | 0.008 | Vermis_7 | 0.035 | 0.018 |
| Precuneus_L | 6.3×10^-4^ | 5.1×10^-4^ | Vermis_3 | 0.016 | 0.021 |

All p>α=2.1×10^-4^, and all β<0 except for vermis_3; with adjustment for rs945270. Models I and II and other abbreviations: same as Table S2.
